# Acute-Onset Blindness in a Patient Diagnosed With Myelin Oligodendrocyte Glycoprotein Antibody Disease (MOG-AD): A Case Report

**DOI:** 10.7759/cureus.61767

**Published:** 2024-06-05

**Authors:** Frederick Gyabaah, Cyrena Petersen, Emily Bateman, Abhizith Deoker

**Affiliations:** 1 Internal Medicine, Texas Tech University Health Sciences Center, El Paso, USA

**Keywords:** mog-igg-associated optic neuritis, sle and lupus nephritis, acute blindness, optic nerve sheath, myelin-oligodendrocyte glycoprotein (mog)

## Abstract

Myelin oligodendrocyte glycoprotein antibody disease (MOG-AD) poses a diagnostic challenge, often masquerading as other neurological disorders such as multiple sclerosis and aquaporin-4-positive neuromyelitis optica spectrum disorder. The deceptive clinical similarities demand a nuanced approach to differentiate these conditions effectively. This entails an extensive evaluation encompassing a meticulous medical history, advanced magnetic resonance imaging (MRI), cerebrospinal fluid analysis, and serum studies. In this context, we present a compelling case involving a 28-year-old Hispanic female with a history of migraine headache. She sought medical attention due to acute peripheral vision loss, ultimately diagnosed as MOG-AD through a comprehensive clinical assessment coupled with specific diagnostic tests. This case underscores the critical importance of precision in diagnostic procedures to ensure accurate identification and subsequent tailored treatment for MOG-AD, avoiding potential pitfalls associated with its resemblance to other neurological disorders.

## Introduction

Myelin oligodendrocyte glycoprotein (MOG) is a glycoprotein found on the outer surface of myelin in the central nervous system (CNS) [1]. It is believed to be involved in the regulation of oligodendrocyte microtubule stability and mediation of the complement cascade. MOG antibody disease (MOG-AD) is a monophasic or relapsing inflammatory demyelinating disease of the CNS associated with the presence of immunoglobulin G (IgG) antibodies against MOG which does not meet the criteria for multiple sclerosis or other neuroinflammatory disorders [2].

Epidemiological data for MOG-AD are limited mainly because MOG-IgG was discovered only in 2007 and widespread testing was not available until years to a decade later. Thus, initial epidemiology reports may have underestimated the frequency of MOG-AD. The incidence and prevalence are largely unknown, although studies in Europe suggest the incidence of MOG-AD is between 1.6 and 3.4 per 1,000,000 person-years. The median age of MOG-AD onset is 20-30 years. Most patients initially present with bilateral optic neuritis (ON) with associated optic nerve head swelling. However, myelitis, aseptic meningitis with leptomeningeal enhancement, acute disseminated encephalomyelitis (ADEM), or an ADEM-like presentation can be a part of the initial presentation as well [3]. There are currently no risk factors known to predispose individuals to the development of MOG-AD. However, it has been associated with demyelinating N-methyl-D-aspartate (NMDA) receptor encephalitis, multiple sclerosis, post-infectious demyelination with herpes simplex virus, and infections with COVID-19, Epstein-Barr virus, or *Borrelia* species. Patients diagnosed with MOG-AD have an increased risk of relapse, around 44-83%, but the residual disability impact and the likelihood of relapse of the disease are still undetermined [4].

This case report details the events of a 28-year-old female with no remarkable family history of autoimmune disorder, who presented with prolonged frontal headache, progressive right-eye vision loss, and papilledema and was diagnosed with MOG-AD. She was treated with acetazolamide, methylprednisolone, and plasmapheresis (PLEX) and made a full recovery.

## Case presentation

We report the case of a 28-year-old Hispanic female with a history of migraine headaches, who initially presented to the emergency department (ED) with concerns of a persistent frontal headache unremitting for five days. The pain was also associated with right-eye vision loss, nausea, and phonophobia. She conveyed that her current symptoms differed from those of her previous migraine episodes. She denied any associated neck pain or stiffness, fever, chills, emesis, shortness of breath, chest pain, or diarrhea. The patient was neurologically intact with no focal deficits. She underwent a computed tomography (CT) scan of the head and a CT angiogram (CTA). Both images were unremarkable. She was discharged from the ED after receiving benefit following treatment with intravenous droperidol 2 mg, hydroxyzine 25 mg, ondansetron 4 mg, and 1 L normal saline bolus.

**Figure 1:**
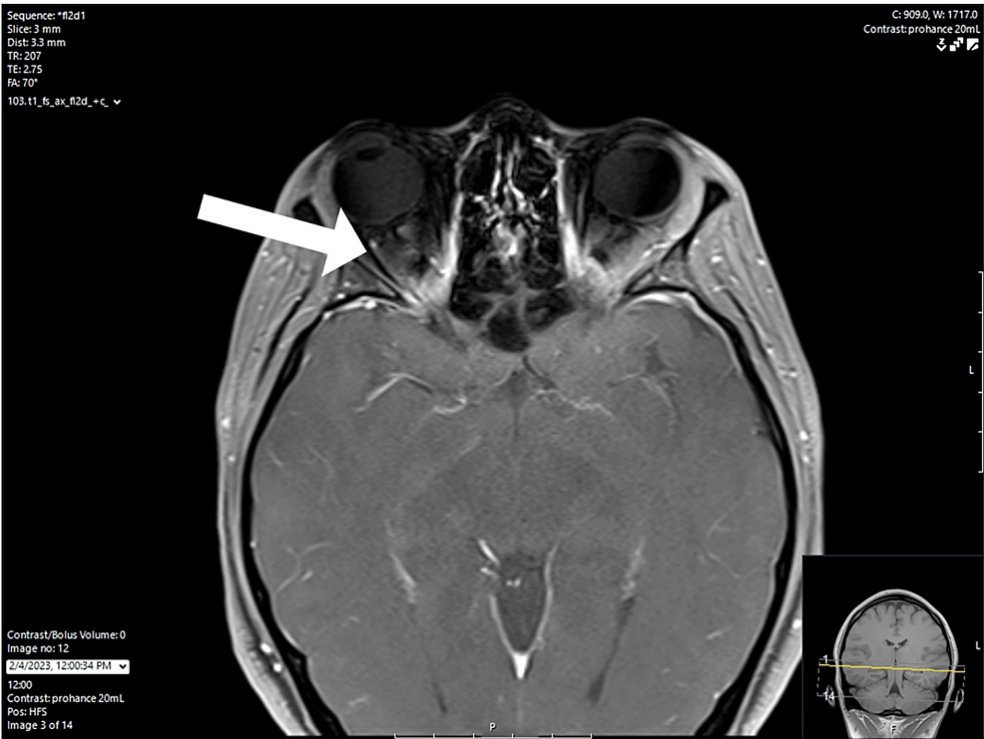
MRI of the face, neck, and orbit with/without contrast MRI: magnetic resonance imaging

**Figure 2:**
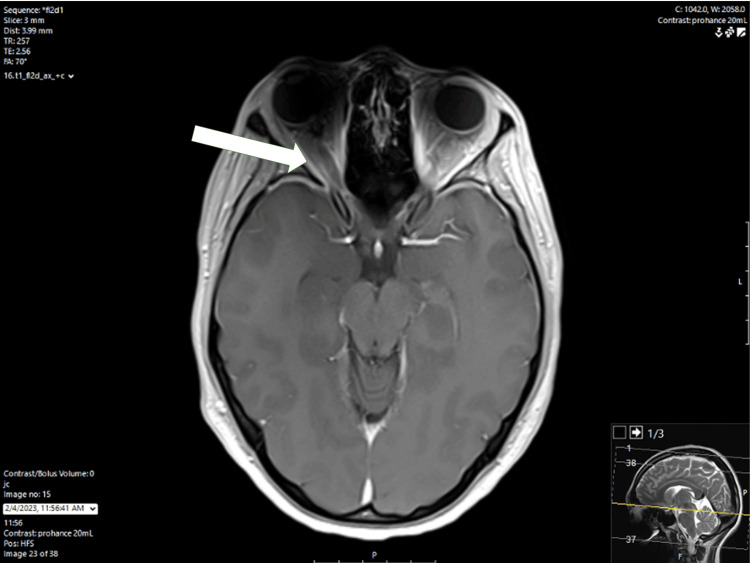
MRI of the brain with/without contrast MRI: magnetic resonance imaging

The patient was prescribed ubrogepant by her primary care physician the next day. Her pain decreased following two doses of this medication; however, the headache still returned shortly after. Five days after the initial ED visit, the patient started to experience decreased peripheral vision in her right eye without ophthalmoplegia. She described this as being able to detect shadows, but unable to see anything else. After two days with these symptoms, she visited a different hospital facility and was admitted for two days, of which records were unavailable for review. On the same day of discharge from that hospital facility, 14 days since the onset of the initial symptoms, she visited an outpatient ophthalmology clinic. Bilateral optic disc edema was found on exam, and she was referred back to the ED for further evaluation of this vision loss and persistent headache. The patient was evaluated in the ED, and a lumbar puncture was attempted but unsuccessful. She was admitted for further evaluation. Magnetic resonance imaging (MRI) of the brain and magnetic resonance angiogram (MRA) of the head and neck were completed on hospital day 1 (HD1) and revealed right greater than left subtle flattening of bilateral posterior globes with edema and enhancement of the right optic nerve head with findings concerning for idiopathic intracranial hypertension (see arrow in Figure 1 and Figure 2).

The patient was started on acetazolamide 250 mg twice daily and oral prednisone 1 mg/kg/day given the MRI findings of possible idiopathic intracranial hypertension. However, the same was discontinued after a lumbar puncture confirmed normal opening pressure and cerebrospinal fluid (CSF) was obtained for analysis. On HD2, the headache resolved; however, her right-sided vision loss persisted. The patient's right eye was fixed and dilated, responsive only to conceptual light, but unable to perceive light response. She was started on methylprednisolone 500 mg every 12 hours for five days. Neuro-ophthalmology was consulted on HD3 and recommended the initiation of PLEX following the third methylprednisolone dose. On HD4, the patient began to have improvement in her right-sided vision but endorsed new-onset abdominal pain and palpitations. A central venous line was placed, and nephrology was consulted to manage PLEX treatments, which would occur once every other day for five treatments.

The patient received her final PLEX treatment on HD12 and was discharged home with visual acuity returned to the reported baseline. Laboratory test results for antibodies were received before discharge, indicating the presence of elevated titers of MOG antibodies. The patient had complete resolution of her symptoms prior to her discharge.

## Discussion

The initial work-up for patients presenting with headache, vision loss, and papilledema includes an MRI of the brain and spinal cord, CSF analysis, and serum studies [4]. The most likely diagnoses include transverse myelitis, acute demyelinating encephalomyelitis, ON secondary to multiple sclerosis, or another autoimmune or inflammatory process [5]. Even though most clinical pictures of this condition are similar to the presentation of neuromyelitis optica spectrum disorder (NMOSD), most experts consider MOG-AD as a distinct entity with different immune system pathology. MOG is a molecule detected on the outer membrane of myelin sheaths and expressed primarily within the brain, the spinal cord, as well as the optic nerve. Multiple studies have shown that many patients referred to ophthalmology care for ON are found to have an alternative diagnosis [6]. One study suggests that nearly 60% of patients referred for ON evaluation were incorrectly diagnosed, with the most common clinical errors being a lack of full differential diagnoses and overreliance on a single portion of the patient history. Although ED diagnostic error rates are oftentimes low, errors still occur, and multiple studies suggest the role of various cognitive biases, such as overconfidence, confirmation, and anchoring [7].

For MOG-AD, the MRI of the brain is abnormal in 45-77% of cases. Findings on MRI can vary, but typically T2-weighted imaging will show poorly demarcated hyperintensities located in three or less locations. Dawson's fingers, U- or S-shaped lesions, and ovoid lesions adjacent to the lateral ventricles can also be present, but are less common [8]. For ON, inflammation is usually in the retro- or intrabulbar part of the optic nerve and a distinctive feature on MRI. However, MOG-AD usually shows more edematous and extensive inflammatory lesions that tend to spare the optic chiasm and tracts when compared to multiple sclerosis-associated or aquaporin-4-positive (AQP4) NMOSD-associated ON. Most MOG-AD patients also have spinal cord T2-weighted hyperintensities localized in the cervical or thoracic regions. However, there was no evidence of such imaging findings on this patient [9]. The CSF shows pleocytosis in most patients and positive oligoclonal bands in some patients. If MOG-AD is suspected, it is important to obtain serum studies for MOG-IgG and aquaporin-4. The aquaporin-4 serum studies are important to obtain to rule out AQP4 NMOSD [10]. The MOG-IgG is the gold standard for diagnosing MOG-AD as it is 99% specific and 100% sensitive for MOG-AD [11,12]. With that being said, it is important to distinguish MOG-AD from the similarly presenting diseases of multiple sclerosis and AQP4 NMOSD as there are differences in treatment protocol and responses. Jarius et al. [13] suggested diagnostic criteria for MOG-IgG testing. The criteria requires evidence of a monophasic or relapsing acute ON, myelitis, and/or encephalitis along with radiological or electrophysiological findings consistent with CNS demyelination accompanied by MRI, fundoscopic, CSF, histopathologic, or clinical findings that are consistent with MOG-AD [14].

Acute treatment of ON and/or myelitis includes the administration of methylprednisolone 1 gram (g) intravenously for five to seven days. If there is an inadequate response to treatment during this time, then PLEX can be considered as a second-line acute treatment, and usually, three to five cycles are administered. It is important to consider that patients with MOG-AD have a high risk for relapse after the completion of steroid treatment, so they require close follow-up and cautious steroid tapering. As such, establishing the correct diagnosis is incredibly important to elucidate the best treatment options for the patient. Without adequate treatment, the disease has high rates of relapse, with a large portion of relapsing patients becoming permanently disabled or functionally blind [15]. Those with rapid succession of relapses have a higher risk of developing generalized demyelinating disease. However, the MOG subset of this disease has been associated with a better prognosis than those who test positive for aquaporin-4 antibodies [16]. Again, patients with persistent MOG-positive antibody are considered to have a poor prognosis for the recurrence of MOG-AD. For these patients, preventative treatment includes oral prednisolone and an oral immunosuppressant, such as azathioprine, mycophenolate, mofetil, or methotrexate, or intravenous immunoglobulins, which requires an induction stage with 2 g/kg followed by 1 g/kg infusions every month [17]. If there is an inadequate response to treatment, then the addition of monoclonal antibodies, such as rituximab, can be considered [18]. Consistent outpatient management through neuro-ophthalmology is an important mainstay of treatment in these individuals.

## Conclusions

This case report detailed the presentation of a 28-year-old Hispanic female who experienced unremitting frontal headaches and visual changes for several days. She visited five different healthcare providers during this time, including a primary care physician, three ED facilities, and an ophthalmologist. Initially, her symptoms were presumed to be migraines by multiple care practitioners. It was only after bilateral optic disc edema was found on exam through ophthalmology that the patient received an MRI. Fortunately, this patient was able to make a full recovery after receiving PLEX and corticosteroids. Early diagnosis of MOG-AD allows for more efficient treatment, which can decrease the risk of permanent deficits and relapses.
